# Exploring the role of CheA3 in *Desulfovibrio vulgaris* Hildenborough motility

**DOI:** 10.3389/fmicb.2014.00077

**Published:** 2014-03-06

**Authors:** Jayashree Ray, Kimberly L. Keller, Michela Catena, Thomas R. Juba, Marcin Zemla, Lara Rajeev, Bernhard Knierim, Grant M. Zane, Jarrod J. Robertson, Manfred Auer, Judy D. Wall, Aindrila Mukhopadhyay

**Affiliations:** ^1^Physical Biosciences Division, Lawrence Berkeley National LaboratoryBerkeley, CA, USA; ^2^Biochemistry Division, University of MissouriColumbia, MO, USA; ^3^Life Sciences Division, Lawrence Berkeley National LaboratoryBerkeley, CA, USA

**Keywords:** sensor histidine kinase, *cheA*, soft agar plate assay, Palleroni chamber assay, electron acceptor, motility

## Abstract

Sulfate-reducing bacteria such as *Desulfovibrio vulgaris* Hildenborough are often found in environments with limiting growth nutrients. Using lactate as the electron donor and carbon source, and sulfate as the electron acceptor, wild type *D. vulgaris* shows motility on soft agar plates. We evaluated this phenotype with mutants resulting from insertional inactivation of genes potentially related to motility. Our study revealed that the *cheA3* (DVU2072) kinase mutant was impaired in the ability to form motility halos. Insertions in two other *cheA* loci did not exhibit a loss in this phenotype. The *cheA3* mutant was also non-motile in capillary assays. Complementation with a plasmid-borne copy of *cheA3* restores wild type phenotypes. The *cheA3* mutant displayed a flagellum as observed by electron microscopy, grew normally in liquid medium, and was motile in wet mounts. In the growth conditions used, the *D. vulgaris ΔfliA* mutant (DVU3229) for FliA, predicted to regulate flagella-related genes including *cheA3*, was defective both in flagellum formation and in forming the motility halos. In contrast, a deletion of the *flp* gene (DVU2116) encoding a pilin-related protein was similar to wild type. We conclude that wild type *D. vulgaris* forms motility halos on solid media that are mediated by flagella-related mechanisms via the CheA3 kinase. The conditions under which the CheA1 (DVU1594) and CheA2 (DVU1960) kinase function remain to be explored.

## Introduction

*Desulfovibrio vulgaris* Hildenborough is an anaerobic model sulfate-reducing bacterium (SRB), representing the broad class of SRB that play an essential role in biogeochemical processes such as sulfur- and metal-cycling (Zhou et al., 2011). Motility, its relation to core physiology such as electron transfer (Tai et al., 2010), and the global nature of its regulation (Ueki et al., 2012) are key topics of research in both model and newly discovered anaerobic metal- and sulfate-reducing organisms (Takaki et al., 2010). The genomes of many organisms that occupy such ecological niches are now sequenced and reveal that some microbes have more than one putative chemotaxis-related gene cluster. *Shewanella oneidensis* MR1 encodes three chemotaxis gene clusters, one of which was shown to respond to electron acceptor concentrations (Bencharit and Ward, 2005; Li et al., 2007). *Geobacter* spp., are also anaerobic metal-reducing bacteria and encode six chemotaxis clusters, the functions of which are yet to be specifically elucidated (Tran et al., 2008).

**Figure 1:**
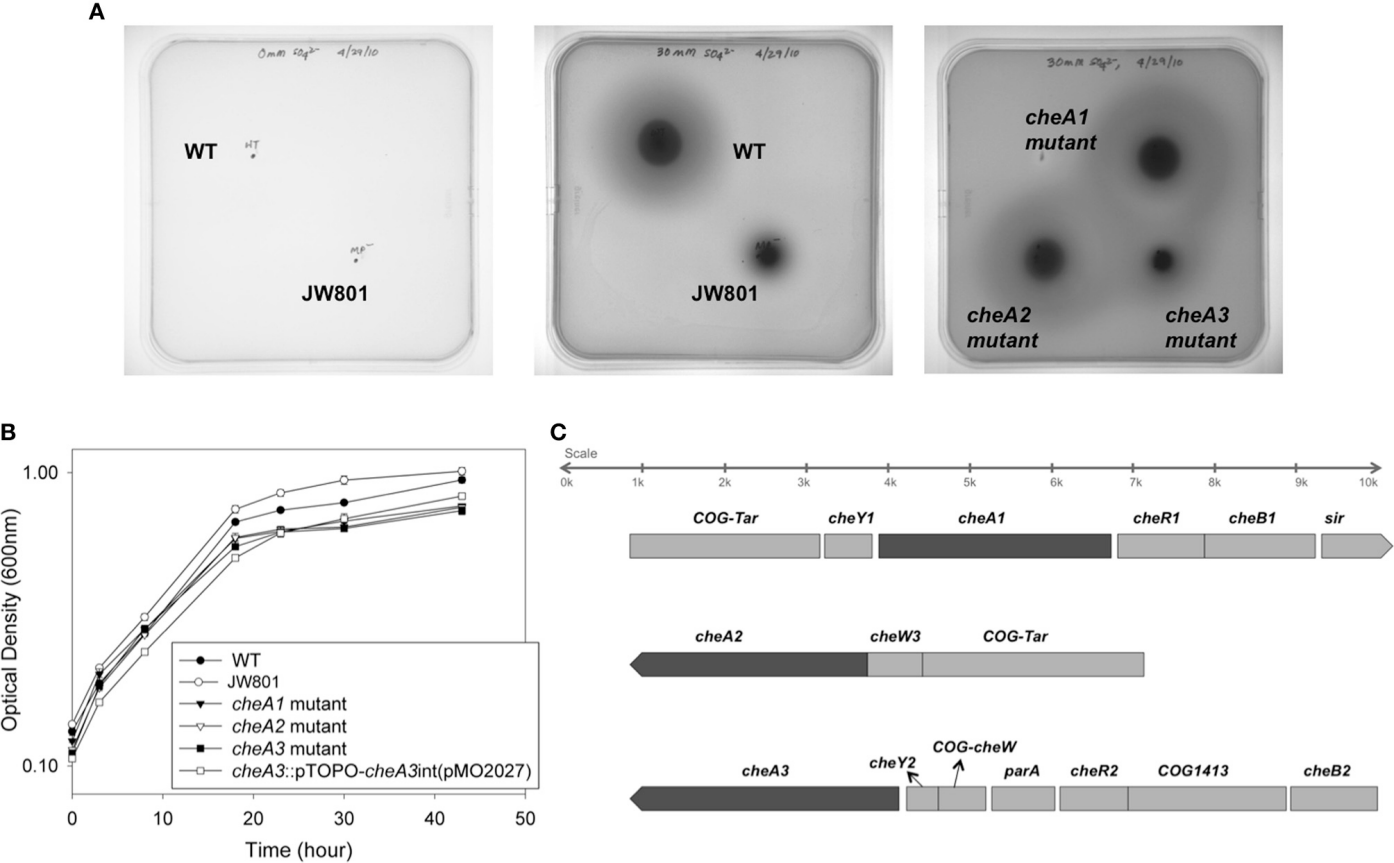
**(A)** Soft agar plate assays for *D. vulgaris* wild type, JW801, CA023 (*cheA1* mutant), CA007 (*cheA2* mutant), and CA022 (*cheA3* mutant) strains in modified LS4D medium as described in methods with 0.4% (wt/vol) agar. Motility halos were imaged after 4 days of incubation in an anaerobic chamber at 30°C. No growth was observed for either the wild type or JW801 strain in the control plate that contains no sulfate (left panel). In LS4D plates, the wild type forms a motility halo, whereas, JW801 was impaired in forming a halo (center panel); the *cheA3* mutant shows a defect in motility (right panel) relative to the *cheA1* and *cheA2* mutants and the wild type. **(B)** Growth assays of *D. vulgaris* wild type, JW801, CA023 (*cheA1* mutant), CA007 (*cheA2* mutant), CA022 (*cheA3* mutant), and the *cheA3* complemented strain, *cheA3::*pTOPO*-cheA3int*(pMO2027). Assays were done in LS4D medium at 30–32°C. Cultures were started at an approximate OD_600_ of 0.1 and grown until the late stationary phase. Data points are the averages of triplicate measurements. **(C)** Operons encoding the three *cheA* chemotaxis genes in *D. vulgaris* as predicted in http://www.microbesonline.org (Dehal et al., 2009). Top: *cheA1*; Middle: *cheA2*; and Bottom: *cheA3*. Arrowheads indicate the direction of transcription.

*D. vulgaris* displays a single polar flagellum (Postgate and Campbell, 1966) and is documented to have motility on soft agar plates prepared with 0.7% (wt/vol) agarose and defined lactate/sulfate medium (Clark et al., 2007), with concentrations not considered limiting for either lactate or sulfate (Postgate, 1963; Mukhopadhyay et al., 2006). Aside from flagellar and pilin protein encoding genes, the genome of *D. vulgaris* encodes three separate chemotaxis clusters, each of which includes a putative *cheA* (Figures 1, S1). Here, we examine the observed motility in *D. vulgaris* as a function of lactate and sulfate in the medium and examine the role of several motility related genes in this phenotype.

## Materials and methods

### Bacterial growth and culture maintenance

All strains and plasmids used in this study are listed in Table 1. *D. vulgaris* Hildenborough strain ATCC 29579 was obtained from the American Type Culture Collection (Manassas, VA, USA). Bacterial strains were grown and maintained as described previously (Mukhopadhyay et al., 2006). Unless noted otherwise, *D. vulgaris* was grown in defined LS4D medium with sodium lactate (60 mM) as the electron donor and sodium sulfate (30 mM) as the electron acceptor. Modified LS4D media, the MOYLS4 and MOY media reported previously (Zane et al., 2010), were used during construction of the *cheA* knock-out mutants. *D. vulgaris* strain JW801, lacking the native plasmid pDV1 (Clark et al., 2007), was used as a non-motile control and was grown similarly to the wild type. For growth of *D. vulgaris* mutant strains CA023, mutated in *cheA1* (DVU1594); CA007, mutated in *cheA2* (DVU1960); and CA022, mutated in *cheA3* (DVU2072). The antibiotic G418 (Sigma Aldrich, St Louis, MO) was added to a final concentration of 400 μg/ml (Keller et al., 2009). For the complementation strain, *cheA3*::pTOPO*-cheA3int*(pMO2027), an additional antibiotic, spectinomycin (100 μg/ml), was added during growth. All *D. vulgaris* stocks were stored in 10% (vol/vol) glycerol at −80°C and were used as 10% (vol/vol) inocula into 10–30 ml of fresh medium and the cells were grown to mid-log phase (optical density at 600 nm (OD_600_) of 0.3 to 0.4).

**Table 1:** **Strains and plasmids used**.

**Strain**	**Description**	**Source**
*D. vulgaris* Hildenborough	Wild type *D. vulgaris* Hildenborough containing the 202 kb plasmid pDV1	ATCC29579
JW801	*D. vulgaris* Hildenborough ΔpDV1	Clark et al., 2007
JW9003	The JW9003 deletion mutant is a deletion of DVU2116 (flp) and DORF39640	This study
JW9017	Δ*fliA* Km^r^	This study
CA007	*cheA2*::pTOPO-*cheA2*int Km^r^	This study
CA022	*cheA3*:: pTOPO-*cheA3*int Km^r^	This study
CA023	*cheA1*:: pTOPO-*cheA1*int Km^r^	This study
GZ10278	*cheA3* Transposon mutant (at bp 2299/3270), *cheA3::*TnRL27	Figueiredo et al., 2013; Zane and Wall, 2013
**PLASMIDS**		
pENTR/D-TOPO	TOPO cloning vector, Km^r^	Invitrogen
pCR2.1-TOPO	TOPO cloning vector, Amp^r^ Km^r^	Invitrogen
pCR8/GW/TOPO	TOPO cloning vector, Spec^r^	Invitrogen
pSC27	*Desulfovibrio* shuttle vector containing the SRB replicon pBG1; source of *aph(3′)-II*; Km^r^	Rousset et al., 1998
pTOPO-cheA3int	Internal 750 bp fragment of *cheA3* cloned into pCR2.1-TOPO Amp^r^ Km^r^	This study
pTOPO-cheA2int	Internal 750 bp fragment of *cheA2* cloned into pCR2.1-TOPO Amp^r^ Km^r^	This study
pTOPO-cheA2int	Internal 750 bp fragment of *cheA1* cloned into pENTR/D-TOPO Km^r^	This study
pMO9002	pCR8/GW/TOPO with 684 bp upstream and 861 bp downstream of *aph(3′)-II* cassette to delete*flp*; Sp^r^ Km^r^	This study
pMO9016	pCR8/GW/TOPO with 960 bp upstream and 942 bp downstream of *aph(3′)-II* cassette to delete *fliA*; Sp^r^ Km^r^	This study
pMO9075	*Desulfovibrio* shuttle vector containing SRB replicon (pBG1) and *aph(3′)-IIp*; Sp^r^; for complementation constructs	Keller et al., 2009
pMO2027	pMO9075 with *aph(3′)-IIp*::*cheA3*; Sp^r^	This study

### Construction of *CheA* insertional mutants

Gene disruption mutants in the *cheA* genes were created by single crossover homologous recombination with suicide vectors containing 750-base pair internal gene regions. The internal gene fragments were produced by PCR amplification with primers listed in Table A1 and cloned into the pENTR/D-TOPO plasmid (Life Technologies, Grant Island, NY, USA). The suicide vectors were confirmed by sequencing and electroporated into wild-type *D. vulgaris* prepared as described previously (Keller et al., 2009). Transformants were recovered and colonies confirmed as described (Zane et al., 2010). Southern blot analysis was performed on all the mutant strains as described previously (Keller et al., 2009) to verify that the gene disruption occurred at the correct locus. The transposon mutant in *cheA3* used for the Palleroni chamber assays (*cheA3*::TnRL27) was obtained from the *D. vulgaris* transposon mutant collection (Zane and Wall, 2013) cited in earlier reports (Fels et al., 2013; Figueiredo et al., 2013; Kazakov et al., 2013).

### Complementation of *CheA*::pTOPO-*CheA3int* mutant

The *cheA3* gene was amplified by Herculase II (Agilent Technologies, Santa Clara, CA, USA) with primers listed in Table A1 and cloned into pMO9075 for expression from the *aph(3')-II* promoter. After selection of the recombinant plasmid and verification of the insert sequence, one isolate was named pMO2027. To obtain a complemented *cheA3* mutant, *cheA3* cells (CA022) were transformed with pMO2027 by electroporation as described (Keller et al., 2009), with the following exceptions: MOYLS4 (60/30, lactate/sulfate) medium was used throughout growth, electroporation, recovery and selective plating of the complemented mutant and the electroporation parameters were set at 1500 V, 250 Ω, and 25 μF. Following sequence verification of the plasmid recovered from the *cheA3* mutant, one isolate was chosen as the complemented strain, *cheA3*::pTOPO*-cheA3int* (pMO2027), for comparison of phenotypes.

### Construction of JW9017 (*fliA*) and JW9003 (*flp*) deletion mutants

The pMO9016 and pMO9002 plasmids for the marker-exchange deletion of *fliA* (DVU3229) and *flp* (DVU2116), respectively, were constructed by splicing by overlap extension (SOE) PCR (Horton et al., 1990) of three PCR amplimers as previously described (Zane et al., 2010). Transformation of the *fliA* and *flp* deletion plasmids into *D. vulgaris* was performed as previously described (Zane et al., 2010), with the exception that the G418-resistant transformants were selected from electroporated cells mixed into molten MOYLS4 medium with 400 μg G418/ml and poured into empty petri dishes for solidification.

### Growth assays

Cells were recovered overnight in 10 ml liquid MOYLS4 medium and used to inoculate 20–25 ml volume of fresh LS4D at a starting OD_600_ of 0.05–0.1. Growth assays were conducted in triplicate under anaerobic conditions at a temperature of 30–32°C. OD_600_ was monitored with a spectrophotometer (Agilent HP Diode Array Model 8452A, Agilent Technologies, Santa Clara, CA, USA) periodically as a function of time until the late stationary phase.

### Soft agar plate assays

**Figure 2:**
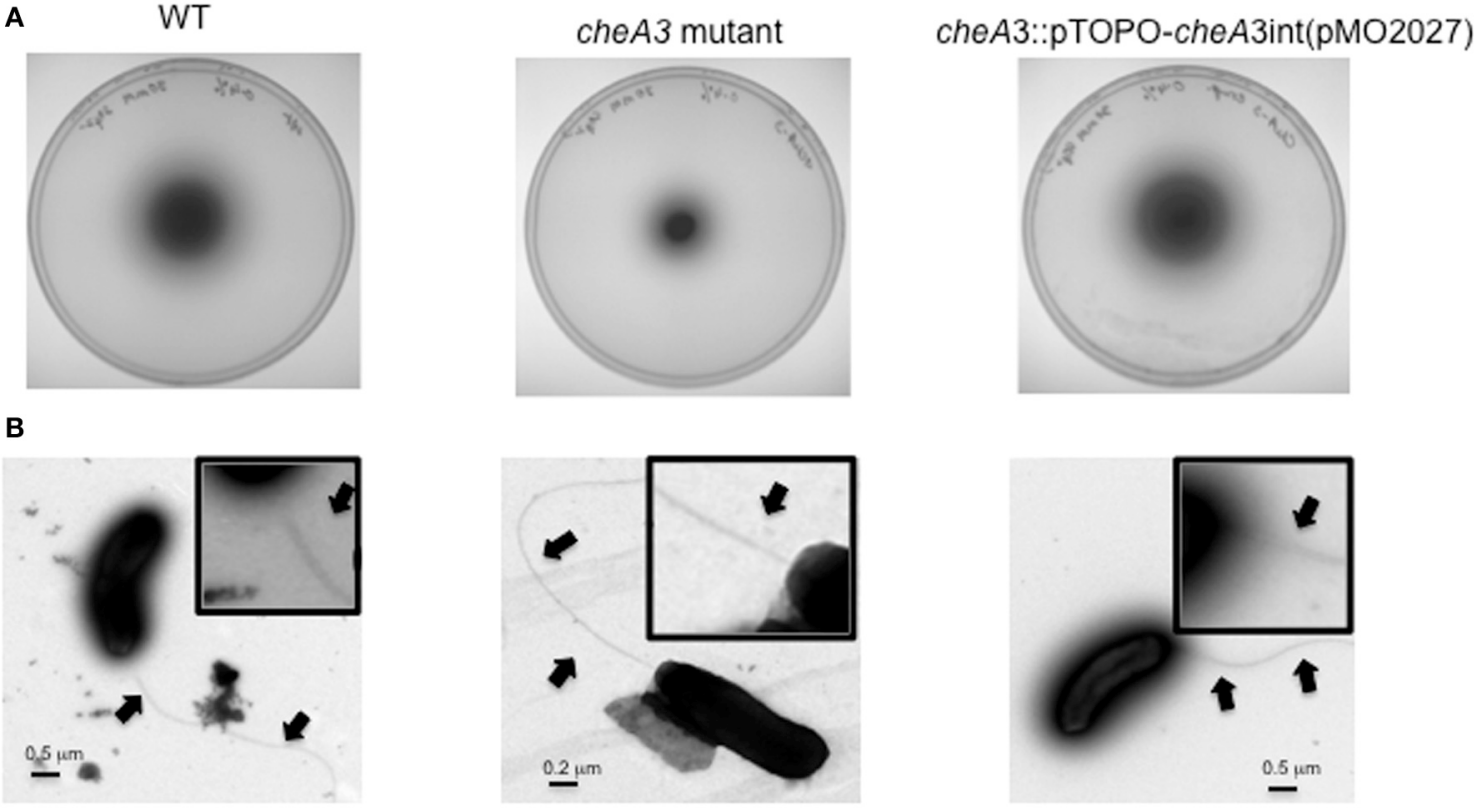
**(A)** Soft agar plate assays of *D. vulgaris* wild type, *cheA3* mutant and *cheA3* complement strain, *cheA3::*pTOPO*-cheA3int*(pMO2027) in LS4D medium with 0.4% (wt/vol) agar and 30 mM sulfate. **(B)** Transmission electron microscopic (TEM) images of the flagella of *D. vulgaris* wild type, *cheA3* mutant and *cheA3* complement strain, *cheA3::*pTOPO*-cheA3int*(pMO2027). In the main images and the enlarged inset views, arrows point to the flagella.

**Figure 3:**
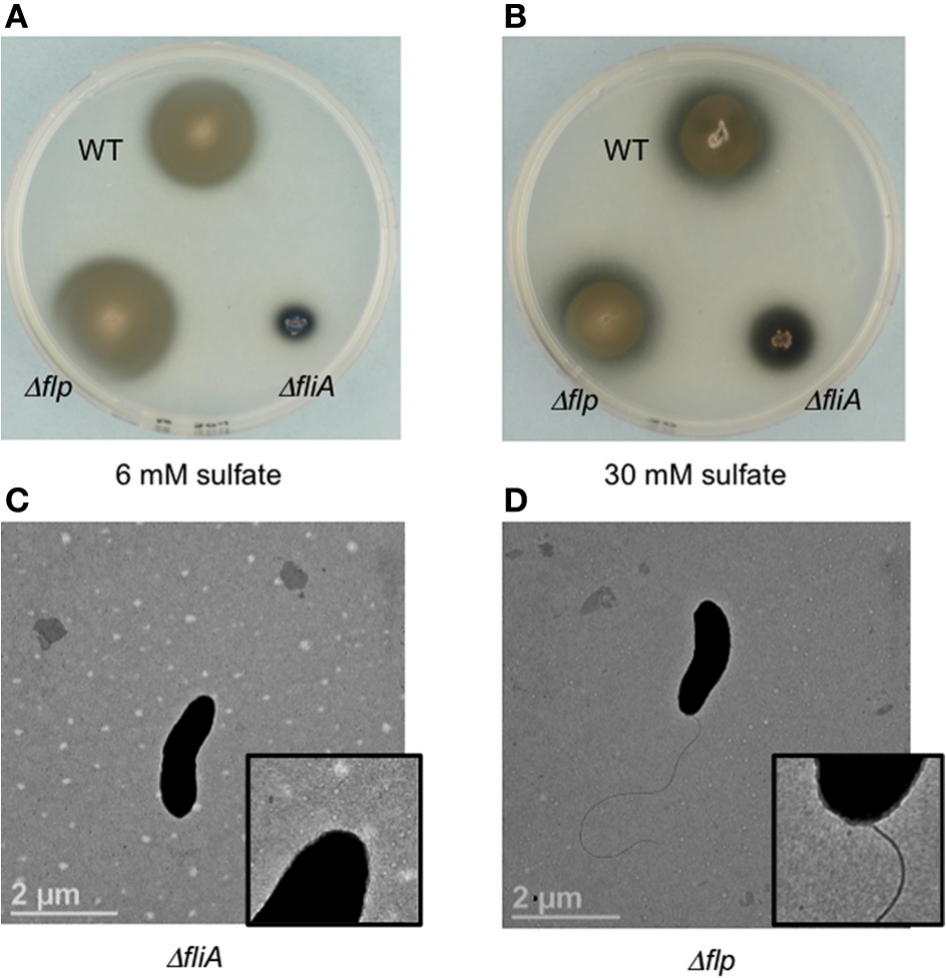
**Soft agar plate assays (0.4% agar wt/vol) of *D. vulgaris* wild type, JW9017 (Δ*fliA)*, and JW9003 (Δ*flp*) in defined LS4D medium with 6 mM sulfate (A) or 30 mM sulfate (B)**. TEM images of JW9017 **(C)** and JW9003 **(D)** grown in defined LS4D medium show the presence of flagellum in the JW9003 strain but not in the JW9017 strain. Inset enlarged views are provided to indicate the flagellum clearly. Note: In rich media, a few cells in JW9017 show the presence of a shorter flagellum (Figure S4).

**Figure 4:**
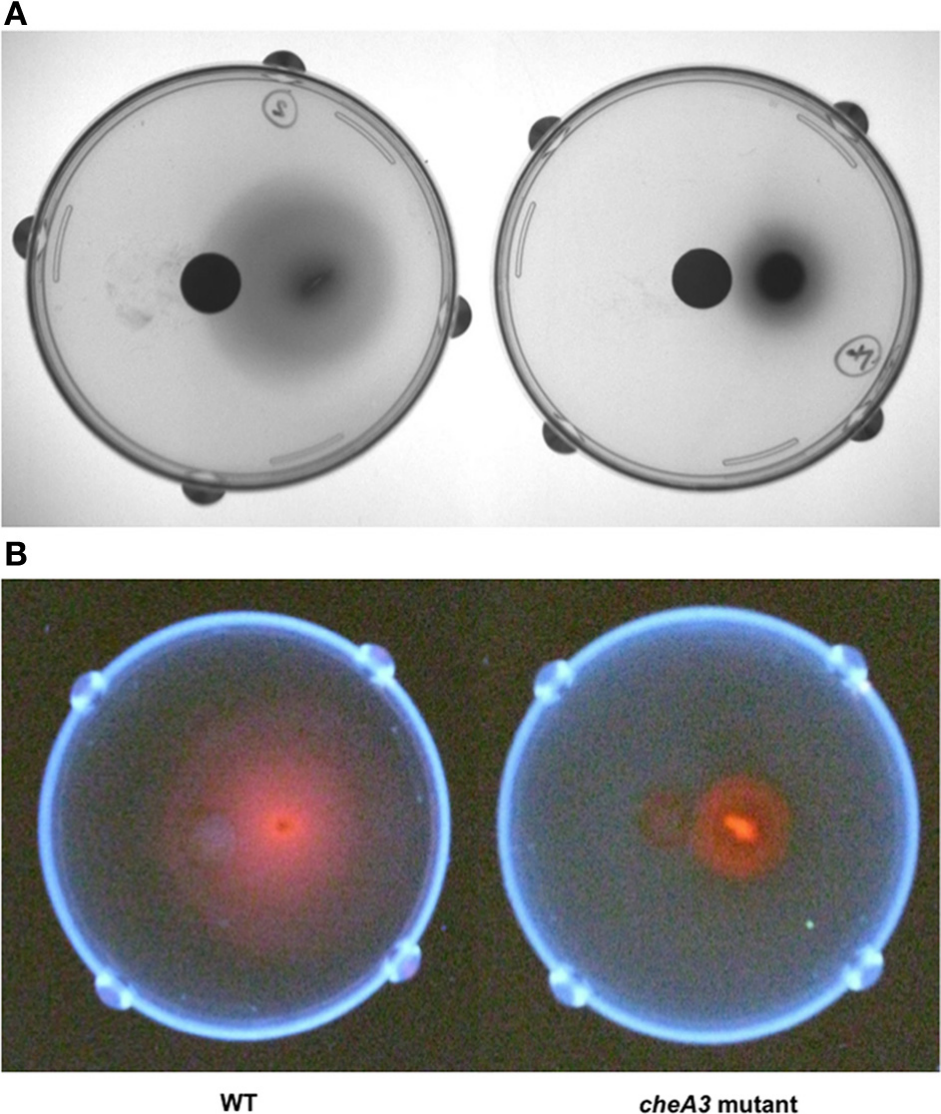
**Soft agar plate disc assays of *D. vulgaris* wild type and *cheA3* mutant strains with a nylon membrane disc soaked in 30 mM sulfate**. Modified LS4D medium contained 0.4% (wt/vol) agar, 12 mM sodium sulfate, and 60 mM sodium lactate. Pictures were taken with white light **(A)** and UV-light **(B)**. Sodium hydroxide solution (5 N) was sprayed over the surface of the agar bed before taking pictures under UV-light to enhance the fluorescence due to the presence of bisulfite reductase containing siroheme as a cofactor (Postgate, 1959).

Soft agar plate assays were used to study the motility as described in other reports (Li et al., 2007) with a few modifications. A modified formulation for LS4D medium was solidified with 0.4% (wt/vol) agar for motility assays. *D. vulgaris* cells were grown to an OD_600_ of 0.3–0.4 and 2 μl of cells were stabbed into the middle of the soft agar bed. For the sulfate disc assays, 0.4% (wt/vol) soft agar medium contained 12 mM sodium sulfate. A nylon membrane disc was pre-soaked in 30 mM sulfate or water and placed 0.5 cm from the center of inoculation immediately prior to inoculation. Plates were incubated at 30–32°C in the anaerobic chamber for 4–5 days to obtain a reasonable amount of motility. Photographs in Figures 1, 2 were taken under white light by a Biospectrum AC Imaging System (UVP, Upland, CA, USA) with the following constant instrumental parameters: exposure time: 634 μs; filter: SyBr Gold (485–655 nm); aperture: 1.2; zoom: 20%; focus: 80%; trans illumination: white. Figure 3A was imaged with a Nikon D5000 camera at the Veterinary Biomedical Communications at the University of Missouri-College of Veterinary Medicine. For the disc assays, images were taken with a white light (Figures 4A, S1) and 365 nm UV-light (Figure 4B) exposure using another UVP imaging system (UVP-chromato-Vue® C-75, UVP, Upland, CA, USA) mounted with a Canon G9 camera. After spraying 5 N sodium hydroxide over the agar bed, *D. vulgaris* cells fluoresce bright pink-orange under the 365 nm UV-light (Figure 4B), which is caused by the release of siroheme, the cofactor of bisulfite reductase desulfoviridin (Postgate, 1959).

### Palleroni chamber assay

A capillary-based assay (Palleroni, 1976) was performed to provide quantitative measurement of the bacterial cell motility, as described previously (Sun et al., 2009). Briefly, 10 ml cultures grown to an OD of approximately 0.4–0.5 (mid-log) were spun down at ~5500 × g for 8 min at room temperature and resuspended in an equal volume of phosphate buffered saline (PBS). Each channel of the Palleroni chamber was filled with 550 μl of resuspended cells. The capillary (32 mm length, 1.1 mm inner diameter) was filled with one of the following solutions: 30 mM sulfate, 60 mM lactate or 1 × PBS (control) and placed horizontally into the Palleroni chamber. After the 15 min incubation period, contents from the capillary were dispensed into 135 μL of 1 × PBS. The micro-bicinchoninic acid (micro-BCA) assay (Pierce, Rockford, IL, USA) was used as per manufacturer's instruction to measure the protein from the cells, and served as a measure of the cell mass that entered the capillary during the assay. Absorbance was measured by the SpectraMax Pro microplate reader (Molecular Devices, Sunnyvale, CA). Dilutions of bovine serum albumin in 1 × PBS were used to prepare a standard curve.

### Electron microscopy

All electron microscopy samples were fixed in 2% (vol/vol) glutaraldehyde (EM grade, purchased from EMS, Hatfield, PA, USA) directly in the growth medium for several hours and then washed in phosphate buffered saline (PBS). For Transmission Electron Microscopy (TEM), a 5 μl sample was put onto a formvar and carbon coated copper grid (200 mesh, Ted Pella, Redding, CA, USA), which was freshly glow-discharged in order to make the carbon film hydrophilic. The sample was allowed to settle for 5 min and the liquid removed with filter paper. Immediately 5 μ l of a 2% (wt/vol) aqueous solution of uranyl acetate was put onto the grid and left for 1 min before also being dried with filter paper. Two quick washes (10 μl each) with distilled water followed. After drying, the grids were investigated with a Phillips Tecnai 12 electron microscope (FEI Company, Hillsboro, OR, USA) with a 120 kV accelerating voltage and magnifications typically between 2900 × and 9300 ×. A Gatan camera (Gatan, Pleasanton, CA, USA) was used for image acquisition.

## Results and discussion

Wild type *D. vulgaris* showed outward motility on soft agar plates over a period of four days relative to the JW801 strain, which lacked the native plasmid pDV1 (Figure 1A). JW801 is known to be non-motile (Clark et al., 2007), possibly due to a defect in flagellum formation, and served as a control. The levels of lactate and sulfate used in these assays were sufficient to permit robust growth of *D. vulgaris* in liquid medium (Postgate, 1963, 1979; Mukhopadhyay et al., 2006).

To investigate a potential role of *cheA* genes in this motility phenotype, gene disruption mutants in all three *cheA* loci were generated and examined on soft agar plates (Figure 1A). The *cheA3* mutant showed a clear defect in this phenotype, whereas the remaining two *cheA* mutants were unaffected. All strains showed similar growth rates and maxima in liquid cultures of LS4D medium (Figure 1B). *cheA3* is the terminal gene in an operon that encodes several chemotaxis genes and genes with other putative functions that have a role in motility (Figure 1C). For example, the *parA* homolog in *Pseudomonas aeruginosa* is known to affect motility, among other phenotypes (Lasocki et al., 2007). Though a polar mutation is unlikely, the *cheA3* gene was complemented in the *cheA3* mutant strain. The complemented mutant, *cheA3::*pTOPO*-cheA3int*(pMO2027), exhibited motility equivalent to the *D. vulgaris* wild type strain (Figure 2A), confirming the direct role of the CheA3 protein in this phenotype. Further, a visual examination of motility on a wet mount at 100 × magnification indicated all three strains to be motile (Supplementary video data). Consistent with this, high resolution TEM (Figure 2B) revealed that all three strains have flagella. Thus loss of motility in the *cheA3* mutant in the soft agar plate is neither correlated with loss of motility in liquid medium nor with a defect in flagellum formation. Taken together, these observations suggest that the wild type motility observed in soft agar LS4D medium plates involve the sensor kinase CheA3 but not CheA1 or CheA2.

The Δ*fliA* mutant, but not a Δ*flp* mutant, was found to be similarly defective in motility halo formation. FliA, a α28 RNA polymerase sigma factor, modulates the formation of the flagellar complex in the model Gram-negative bacterium *Escherichia coli* (Komeda, 1986). *D. vulgaris* also contains a *fliA* homolog (DVU3229), encoding a σ^70^ transcription factor, that is predicted to modulate 16 genes, including genes involved in the formation of the flagellum and *cheA3* (Novichkov et al., 2010). The Δ*fliA* mutant (strain JW9017) was used to examine the role of the flagellum in the motility halo formation. In 0.4% (wt/vol) agar plates, this strain was severely impaired in halo formation (Figures 3A,B). TEM images of the FliA mutant confirmed it to be defective in flagellum formation (Figure 3C). Unlike the *cheA3* mutant strain, the FliA mutant is non-motile as observed on wet mounts (data not shown). *D. vulgaris* also encodes genes for pilin formation, such as a putative *flp* gene (DVU2116) (Heidelberg et al., 2004). Flp pili are typically not known to mediate twitching motility and the *D. vulgaris Δflp* mutant (strain JW9003), when tested on 0.4% (wt/vol) soft agar plates showed no defect in the motility halo forming phenotype (Figures 3A,B). TEM images also show that the Δ*flp* strain displays the polar flagellum (Figure 3D). While more characterization is required to confirm the motility mode leading to the halos in *D. vulgaris*, the evidence points toward a flagellum-based mechanism.

**Figure 5:**
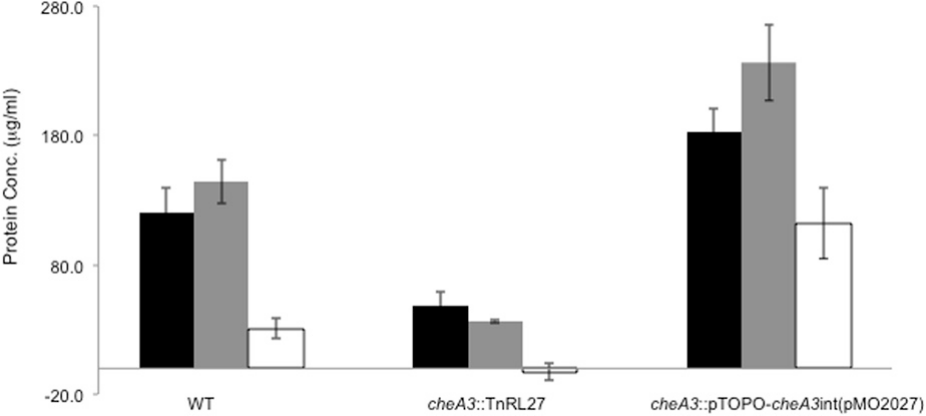
**Palleroni chamber assay to examine the accumulation of cells in a capillary tube containing either 30 mM sulfate (black bar), 60 mM lactate (gray bar), or PBS (white bar) for the wild type, *cheA3* mutant *cheA3::*TnRL27 and the *cheA3* complement *cheA3::*pTOPO*-cheA3int*(pMO2027) strains**. Assays were conducted in triplicate. Error bars are standard deviation of the means.

Upon using two different concentrations of sulfate in the soft agar plates, we observed larger motility halos for the lower concentration of sulfate (Figures 3A,B) in both the wild type and the Δ*flp* mutant. In order to evaluate a possible correlation of *D. vulgaris* motility with sulfate, we performed two assays. First, we used a soft agar plate assay with a nylon membrane disc soaked in sulfate as described in the methods. An asymmetric motility of wild type *D. vulgaris* was observed toward the sulfate-soaked disc (Figure 4). Neither the halo nor the asymmetry was observed for the *cheA3* mutant (Figure 4). The asymmetry was also not observed in the wild type *D. vulgaris* with either water-soaked (Figure S2) or lactate-soaked discs (Figure S3). Second, we conducted a Palleroni chamber-based assay, specifically used to test for swim-related phenotypes (Palleroni, 1976; Sun et al., 2009). For both the wild type and the *cheA3* complemented strain, we observed a similar and significantly greater accumulation of cells in the capillary with lactate and sulfate, relative to the control (PBS) (Figure 5). The capillary assay results corroborate the ability of the wild type *D. vulgaris* to move toward sulfate and, unlike the plate-based assays, also toward lactate. Additional experiments will be required to examine the differences in *D. vulgaris* wild type motility toward lactate, between the soft agar plate assays and the capillary assays. Finally, consistent with the plate-based assays, the accumulation of cells for the *cheA3* mutant was significantly lower in the conditions tested (Figure 5). Thus the *cheA3* mutant may be generally impaired in directional motility.

Possible causes for the observed outward motility on soft agar plates could be toward a nutrient as it gets depleted or away from an inhibitory compound that gets deposited during growth. The taxis observed toward nutrients in the capillary assay suggest the former to be the case. Terminal electron acceptors are known to be limiting in freshwater environments that SRB occupy (Hazen and Tabak, 2005). In *Desulfovibrio* spp., reports exist for aerotaxis (Eschemann et al., 1999), where some species have been shown to move toward low levels of oxygen (Fischer and Cypionka, 2006), and have even been postulated to use low levels of O_2_ as an electron acceptor (Cypionka, 2000). For *D. vulgaris* Hildenborough specifically, established electron acceptors supporting growth are sulfate (Postgate, 1963), thiosulfate and sulfite (Heidelberg et al., 2004). Even though *D. vulgaris* has been reported to reduce transition group metals such as iron, strontium, chromium and uranium (Lovley and Phillips, 1994; Payne et al., 2002; Park et al., 2008), sustained growth has not been reported during reduction of these metals (Payne et al., 2002; Park et al., 2008). As in *Desulfovibrio* spp., multiple chemotaxis modules are known to exist in many other bacteria, including *Geobacter* spp. (Tran et al., 2008), *Vibrio cholerae* (Gosink et al., 2002), *P. aeruginosa* (Kato et al., 1999), *Rhodobacter sphaeroides* (Gauden and Armitage, 1995; Martin et al., 2001), and *M. xanthus* (Yang et al., 1998). Where characterized, such as in *S. oneidensis*, only one CheA is responsible for movement toward electron acceptors (Bencharit and Ward, 2005; Li et al., 2007). Taken together, our results indicate that CheA3 may play this role in *D. vulgaris* Hildenborough. Homologs of the *cheA3* in related bacteria (Figure S1), such as *D. vulgaris* Miyazaki and *D. alaskensis* G20, probably also perform the same function. As gene deletion mutant libraries become available in these bacteria, it will be possible to experimentally verify these predictions.

### Conflict of interest statement

The authors declare that the research was conducted in the absence of any commercial or financial relationships that could be construed as a potential conflict of interest.

## Supplementary material

Figure S1 **The *cheA3* histidine kinase operon is conserved between closely and distantly related *Desulfovibrio* species and few other organisms**. Figure obtained from microbesonline.org (Dehal et al., 2009).

Figure S2 **Control assay for data presented in Figure 4**. Soft agar plate disc assays of *D. vulgaris* wild type with a nylon membrane disc soaked in water. Modified LS4D medium in the agar contained 0.4% (wt/vol) agar, 12 mM sodium sulfate, and 60 mM sodium lactate.

Figure S3 **Soft agar plate disc assays of *D. vulgaris* wild type with a nylon membrane disc soaked in 60 mM lactate**. Modified LS4D medium in the agar contained 0.4% (wt/vol) agar, 10 mM sodium lactate, and 30 mM sodium sulfate.

Figure S4 **TEM images of JW9017 mutant (lacking *fliA*) in rich media**. Arrows are used to label possible truncated flagellum.

Supplementary video data **Video recording of *D. vulgaris* wild type, *cheA3* mutant and *cheA3* complement strain, *cheA3::*pTOPO*-cheA3int*(pMO2027) in wet mounts using a Samsung galaxy camera-phone held at the eye piece of a Leica DM4000 microscope at 100x magnification**.

## Appendix

**Table A1:** **Primers used for Southerns and Sequencing verification**.

**Target gene**	**Primer name**	**Sequence 5′ to 3′**	**Use**
*cheA3*	P1	CCAAGCTTAGGAGACGAACGAAGTTTCCGTCGACCTGCAGCGGAATTCGCAGCGGCCTGCGACCCCTC	Amplification of internal 750 bp fragment of* cheA1* to generate suicide insertion vector (sense)
*cheA3*	P2	CCGGATCCGTAGTCGTACTCATGCTGACCGAGCTCGAATTCAGAATTCGGGGGCCGGGGCGGCGGGAC	Amplification of internal 750 bp fragment of* cheA1* to generate suicide insertion vector (antisense)
*cheA1*	P3	CCAAGCTTCTATGCTACACCGCAGAGGAGTCGACCTGCAGCGGAATTCCGATGCGACCGTTGATGTGC	Amplification of internal 750 bp fragment of* cheA2* to generate suicide insertion vector (sense)
*cheA1*	P4	CCGGATCCGCGCACCTACGACGGTTATACGAGCTCGAATTCAGAATTCATGGTCACCAGCACCTCGCC	Amplification of internal 750 bp fragment of* cheA2* to generate suicide insertion vector (antisense)
*cheA2*	P5	CCAAGCTTACGCCGTAACACGTACATAGGTCGACCTGCAGCGGAATTCACCGGCCGGGTCTCTGCTGA	Amplification of internal 750 bp fragment of *cheA3* to generate suicide insertion vector (sense)
*cheA2*	P6	CCGGATCCAGGCACAGAACCGATCACGTCGAGCTCGAATTCAGAATTCAAGGTCTACCCCGGCACCGT	Amplification of internal 750 bp fragment of *cheA3* to generate suicide insertion vector (antisense)
*cheA3*	P7	ATGACTCAGGAATATATGGATCCGGAAATATTCG	Amplification of *cheA3* to generate complementation vector
*cheA3*	P8	TCATATGGCCTTGGAAGTGGCCAT	Amplification of *cheA3* to generate complementation vector
	P9	GCTGAAAGCGAGAAGAGCGCAC	Amplification of insert from pMO2072 for sequencing
	P10	TGGGTTCGTGCCTTCATCCG	Amplification of insert from pMO2072 for sequencing
	P11	CAAGGATCTGATGGCGCAGGG	Amplification of pMO9075 backbone for construction of pMO2027
	P12	CTGGGACTGCATTGCAGGGCTTCCCAACCT	Amplification of pMO9075 backbone for construction of pMO2027
*cheA1*	P13	AACGACGGCCAGTCTTAAGC	Amplification of insert from pENTR/D-TOPO for probe creation for Southern blot analysis
*cheA1*	P14	AGACACGGGCCAGAGCTG	Amplification of insert from pENTR/D-TOPO for probe creation for Southern blot analysis
*cheA2 cheA3*	P15	GAC CGG CAG CAA AAT G	Amplification of insert from pCR2.1 TOPO for probe creation for Southern blot analysis, and sequence confirmation.
*cheA2 cheA3*	P16	CAG GAA ACA GCT ATG AC	Amplification of insert from pCR2.1 TOPO for probe creation for Southern blot analysis, and sequence confirmation.
	P17	AACGTCGACAAGGCGACACTG	Amplification of region upstream of *flp*
	P18	**AAGACTGTAGCCGTACCTCGAATCTA** TGTGTGCCTCGTTGGCTGC	Amplification of region upstream of *flp*
	P19	**AATCCGCTCACTAAGTTCATAGACCG** CACCAATCCCGACGGACC	Amplification of region downstream of *flp*
	P20	CAGTGCCGCTATGACCTGTAT	Amplification of region downstream of *flp*
*aph(3′)-II*	P21	**TAGATTCGAGGTACGGCTACAGTCTT** *ACCTAGCAACAGAGACCGTG* CCCCAGAGTCCCGCTCAG	Amplification of *aph(3′)-II* cassette for the *flp* deletion cassette with common and unique barcodes
*aph(3′)-II*	P22	**CGGTCTATGAACTTAGTGAGCGGATT***GTGACGTGACCTGATGACTA* GAGGTAGCTTGCAGTGGGCT	Amplification of *aph(3′)-II* cassette for the *flp* deletion cassette with common and unique barcodes
	P23	GCTGGTCTTCAAGCGCCAGTT	Amplification of region upstream of *flp*
	P24	AAGACTGTAGCCGTACCTCGAATCTA CCAGAGCCGCCGGAAC	Amplification of region upstream of *fliA*
	P25	AATCCGCTCACTAAGTTCATAGACCG CACAGCGTGCAAGGAGCC	Amplification of region downstream of *fliA*
	P26	GCGAACTTGCACACCAGAAAGC	Amplification of region downstream of *fliA*
*aph(3′)-II*	P27	**TAGATTCGAGGTACGGCTACAGTCTT***GAACTGGTGAGACCGACCTA* CCCCAGAGTCCCGCTCAG	Amplification of *aph(3′)-II* cassette for the *fliA* deletion cassette with common and unique barcodes
*aph(3′)-II*	P28	**CGGTCTATGAACTTAGTGAGCGGATT** *CACCTGTAACTACTACTAGG* GAGGTAGCTTGCAGTGGGCT	Amplification of *aph(3′)-II* cassette for the *fliA* deletion cassette with common and unique barcodes
	P29	GTTGCAACAAATTGATGAGCAATGC	Screening for clones and sequencing of pMO9002 and pMO9016
	P30	GTTGCAACAAATTGATGAGCAATTA	Screening for clones and sequencing of pMO9002 and pMO9016
	P31	CTCATCCTGTCTCTTGATCAGATCT	Sequencing of pMO9002 and pMO9016 out of Km cassette
	P32	CTACCCGTGATATTGCTGAAGAG	Sequencing of pMO9002 and pMO9016 out of Km cassette
	P33	GGC ACG TCA CGC CCA TCT	Sequencing of pMO9002
	P34	AGA TGG GCG TGA CGT GCC	Sequencing of pMO9002
	P35	AAC TGG CTC ACC TTT CCG GC	Sequencing of pMO9002
	P36	GCC GGA AAG GTG AGC CAG TT	Sequencing of pMO9002
	P37	GGC ACG TCA CGC CCA TCT	Sequencing of pMO9016
	P38	AGA TGG GCG TGA CGT GCC	Sequencing of pMO9016
	P39	AAC TGG CTC ACC TTT CCG GC	Sequencing of pMO9016
	P40	GCC GGA AAG GTG AGC CAG TT	Sequencing of pMO9016

**Sequences** represent the common barcode sequences. Sequences represents the unique barcode sequences.
