# Supplementary figures and images for: Exploring the role of CheA3 in Desulfovibrio vulgaris Hildenborough motility
Source: Front Microbiol. 2014 Mar 6;5:77. doi: 10.3389/fmicb.2014.00077 (PMC3944678; doi:10.3389/fmicb.2014.00077)

Fig. S2

WT *D. vulgaris* Hildenborough

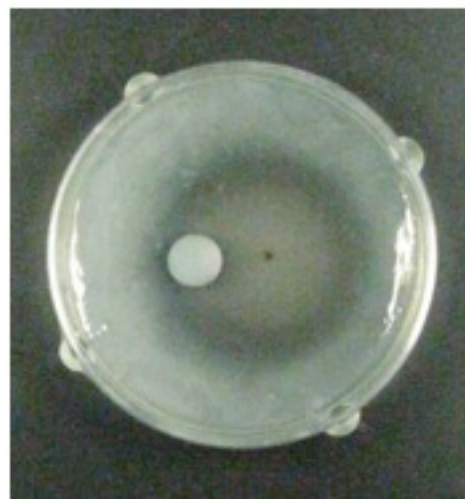

Supplement: Figure S2 — Control assay for data presented in Figure 4. Soft agar plate disc assays of D. vulgaris wild type with a nylon membrane disc soaked in water. Modified LS4D medium in the agar contained 0.4% (wt/vol) agar, 12 mM sodium sulfate, and 60 mM sodium lactate. [file Presentation2.PDF]

Fig. S3

WT *D. vulgaris* Hildenborough

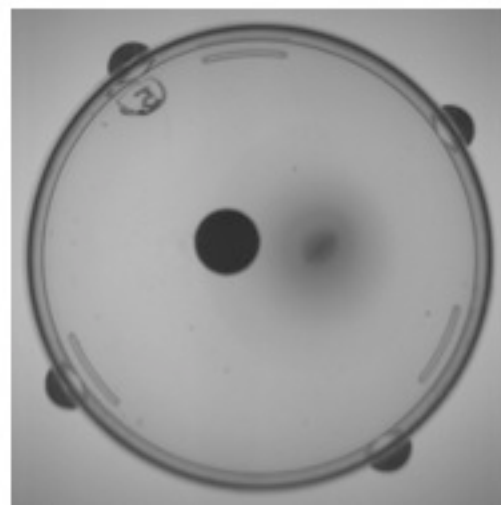

Supplement: Figure S3 — Soft agar plate disc assays of D. vulgaris wild type with a nylon membrane disc soaked in 60 mM lactate. Modified LS4D medium in the agar contained 0.4% (wt/vol) agar, 10 mM sodium lactate, and 30 mM sodium sulfate. [file Presentation3.PDF]

Fig. S4

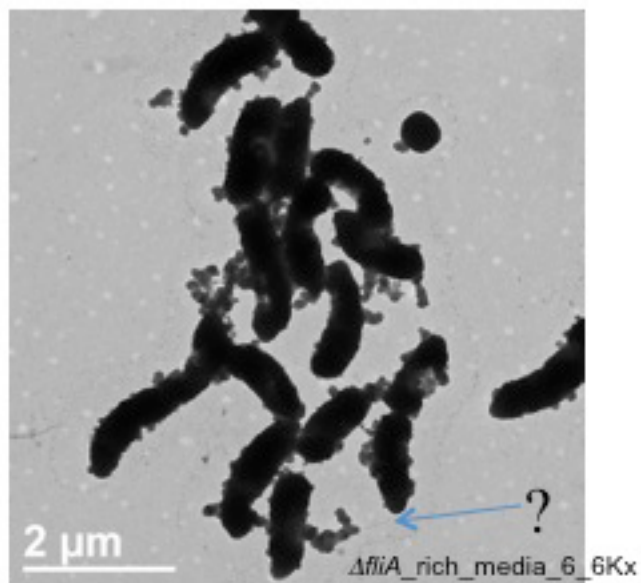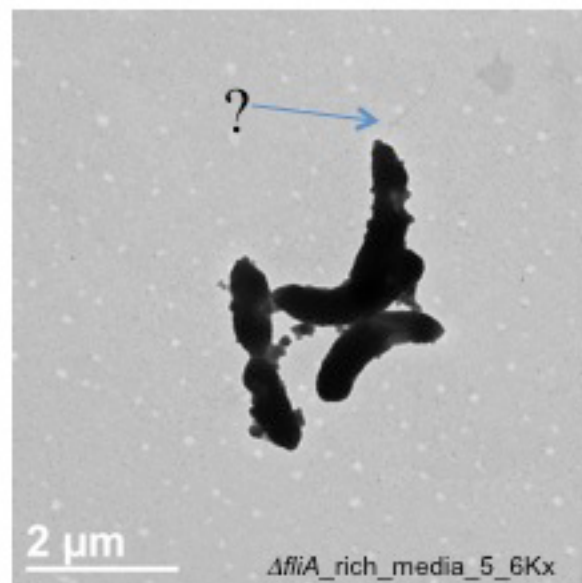

Supplement: Figure S4 — TEM images of JW9017 mutant (lacking fliA) in rich media. Arrows are used to label possible truncated flagellum. [file Presentation4.PDF]
