# Supplementary material for: Exploring the role of CheA3 in Desulfovibrio vulgaris Hildenborough motility
Source: Front Microbiol. 2014 Mar 6;5:77. doi: 10.3389/fmicb.2014.00077 (PMC3944678; doi:10.3389/fmicb.2014.00077)
Supplement: Figure S1 — The cheA3 histidine kinase operon is conserved between closely and distantly related Desulfovibrio species and few other organisms. Figure obtained from microbesonline.org (Dehal et al., 2009). [file Presentation1.PDF]

Fig. S1

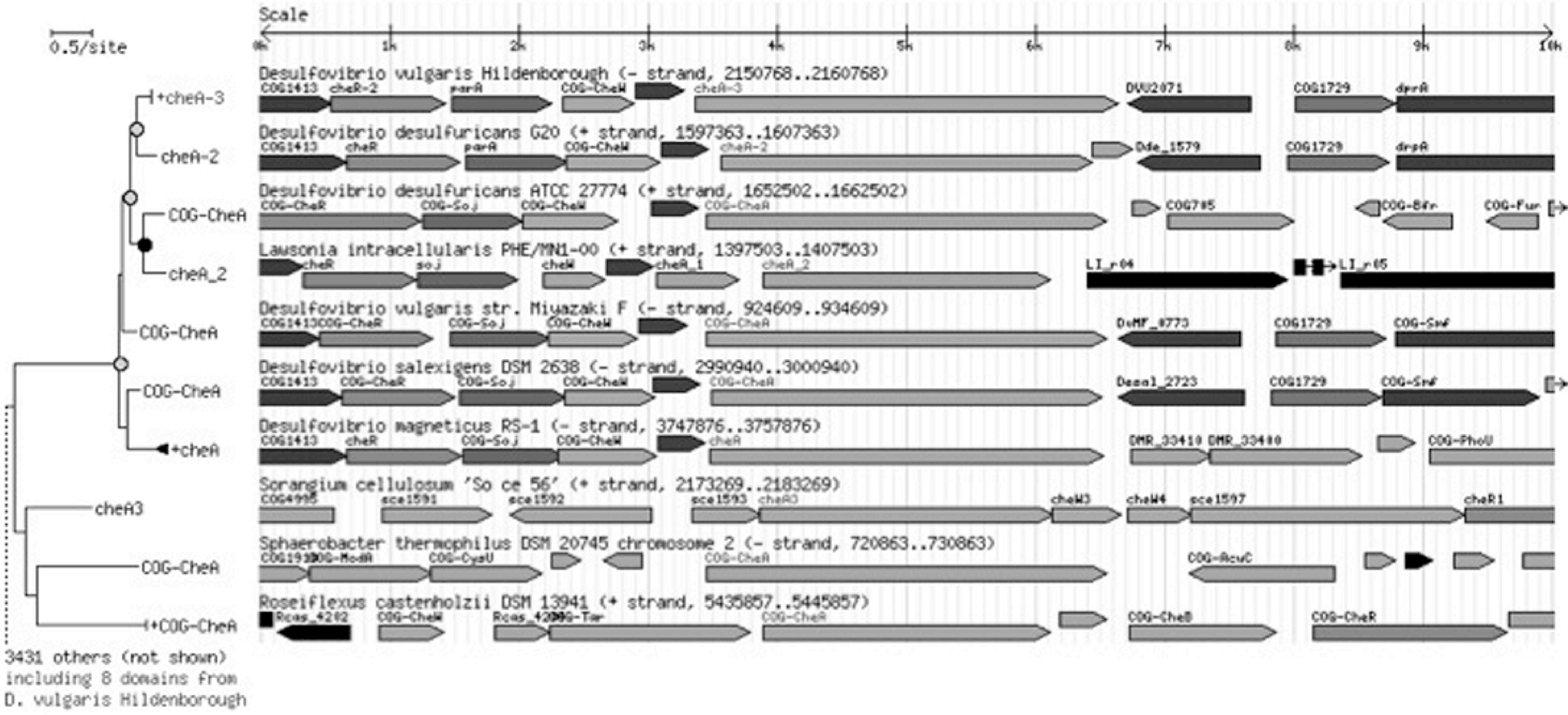

Scale
0.5/site
0k
1k
2k
3k
4k
5k
6k
7k
8k
9k
10k
+cheA-3
cheA-2
COG-CheA
cheA_2
COG-CheA
COG-CheA
+cheA
cheA3
COG-CheA
+COG-CheA
Desulfovibrio vulgaris Hildenborough (- strand, 2150768..2160768)
COG1413 cheR-2 parA COG-CheW cheA-3 DVU2071 COG1729 dpsA
Desulfovibrio desulfuricans G20 (+ strand, 1597363..1607363)
COG1413 cheR parA COG-CheW cheA-2 Dde_1579 COG1729 dpsA
Desulfovibrio desulfuricans ATCC 27774 (+ strand, 1652502..1662502)
COG-CheR COG-Soj COG-CheW COG-CheA COG-Bfr COG-Fur
Lawsonia intracellularis PHE/MN1-00 (+ strand, 1397503..1407503)
cheR soj cheW cheA_1 cheA_2
Desulfovibrio vulgaris str. Miyazaki F (- strand, 924609..934609)
COG1413 COG-CheR COG-Soj COG-CheW COG-CheA COG1729
Desulfovibrio salexigens DSM 2638 (- strand, 2990940..3000940)
COG1413 COG-CheR COG-Soj COG-CheW COG-CheA Desal_2723 COG1729
Desulfovibrio magneticus RS-1 (- strand, 3747876..3757876)
COG1413 cheR COG-Soj COG-CheW cheA COG-PhoU
Sorangium cellulosum 'So ce 56' (+ strand, 2173269..2183269)
sce1591 sce1592 sce1593 cheA3 cheW3 cheW4 sce1597 cheR1
Sphaerobacter thermophilus DSM 20745 chromosome 2 (- strand, 720863..730863)
COG-ModA COG-CysU COG-CheA COG-AcuC
Roseiflexus castenholzii DSM 13941 (+ strand, 5435857..5445857)
Rcas_4202 COG-CheW COG-CheA COG-CheB COG-CheR
3431 others (not shown)
including 8 domains from
D. vulgaris Hildenborough
